# Chemical Defense of the Eastern Newt (*Notophthalmus viridescens*): Variation in Efficiency against Different Consumers and in Different Habitats

**DOI:** 10.1371/journal.pone.0027581

**Published:** 2011-12-02

**Authors:** Zachary H. Marion, Mark E. Hay

**Affiliations:** 1 School of Biology, Georgia Institute of Technology, Atlanta, Georgia, United States of America; 2 Department of Ecology and Evolutionary Biology, University of Tennessee, Knoxville, Tennessee, United States of America; Institute of Marine Research, Norway

## Abstract

Amphibian secondary metabolites are well known chemically, but their ecological functions are poorly understood—even for well-studied species. For example, the eastern newt (*Notophthalmus viridescens*) is a well known secretor of tetrodotoxin (TTX), with this compound hypothesized to facilitate this salamander's coexistence with a variety of aquatic consumers across the eastern United States. However, this assumption of chemical defense is primarily based on observational data with low replication against only a few predator types. Therefore, we tested the hypothesis that *N. viridescens* is chemically defended against co-occurring fishes, invertebrates, and amphibian generalist predators and that this defense confers high survivorship when newts are transplanted into both fish-containing and fishless habitats. We found that adult eastern newts were unpalatable to predatory fishes (*Micropterus salmoides, Lepomis macrochirus*) and a crayfish (*Procambarus clarkii*), but were readily consumed by bullfrogs (*Lithobates catesbeianus*). The eggs and neonate larvae were also unpalatable to fish (*L. macrochirus*). Bioassay-guided fractionation confirmed that deterrence is chemical and that ecologically relevant concentrations of TTX would deter feeding. Despite predatory fishes rejecting eastern newts in laboratory assays, field experiments demonstrated that tethered newts suffered high rates of predation in fish-containing ponds. We suggest that this may be due to predation by amphibians (frogs) and reptiles (turtles) that co-occur with fishes rather than from fishes directly. Fishes suppress invertebrate consumers that prey on bullfrog larvae, leading to higher bullfrog densities in fish containing ponds and thus considerable consumption of newts due to bullfrog tolerance of newt chemical defenses. Amphibian chemical defenses, and consumer responses to them, may be more complex and indirect than previously appreciated.

## Introduction

Predation plays a key role in determining the composition and structure of communities [1]–[3]. Consumers often dictate the realized niches of prey [4]–[6], with predators affecting prey distributions and abundances through both direct consumption [7] and through fear and intimidation [8], [9].

In lentic freshwater habitats, the influences of predation and hydroperiod create a habitat gradient ranging from transient pools with few predators to permanent lakes and reservoirs with abundant consumers [10]. Large consumers, such as fishes, that are capable of facilitating top-down trophic cascades are often excluded from ephemeral environments by periodic drying events [11]. As water permanence increases, so do the densities and diversity of predators [10] . In the most permanent aquatic habitats, fishes are usually present and are often critically important predators that govern the distribution and abundance of many prey species [12], [13]. Notably, fishes often impose a selective sieve that limits undefended, active, and rapidly developing prey species to ephemeral habitats while selecting for defended, less active, and slower developing species that are less prone to predator exclusion [10], [11], [14].

Yet prey are not passive players in the ecological and evolutionary game; selection by consumers has led to a diverse array of morphological (e.g., crypsis [15]), behavioral (e.g., death feigning [16]), or chemical (e.g., toxic skin secretions [17], [18]) adaptations to avoid or deter consumers. Such adaptations allow prey species to coexist with consumers, creating a mosaic of species distributions that vary temporally and spatially with consequences for population, community, and ecosystem-level processes [5], [19].

Although chemical defenses can affect biotic interactions with effects that cascade from individuals through to entire ecosystems [20], our knowledge of chemically-mediated prey defenses and their ecological and evolutionary impacts in freshwater systems is limited compared to chemically mediated interactions in terrestrial and marine systems [21]. Additionally, chemical ecology is a relatively new field; most of the attention thus far has focused on plants and their herbivores or on plant-like, sessile marine invertebrates (e.g., tunicates, sponges) and their consumers (e.g., [20], [22]). Chemical defenses among mobile species occupying higher trophic levels have received less consideration [23], [24].

Among vertebrates, amphibians are the most notable and diverse taxonomic group using putative chemical defenses [17]. Many amphibians secrete a pharmacopoeia of noxious compounds (e.g., alkaloids, amines, peptides, steroids) with a variety of physiological effects (see [17], [25], [26] for reviews). Dramatic examples of toxic amphibians include the rough-skinned newt (*Taricha granulosa*), a salamander species capable of harboring enough tetrodotoxin (TTX) per individual to kill 25,000 mice if the compound is injected [27], [28]; this newt serves as the model to the Batesian mimic *Ensatina eschscoltzii xanthoptica* [29]. The skin of *Phyllobates terribilis*, the golden dart frog, contains up to 1.9 mg of the cardiotoxic and neurotoxic batrachotoxin, one of the most poisonous natural compounds known [30]. Still, both *T. granulosa* and *P. terribilis* are readily consumed by a few well-adapted predators [30], [31]. However, despite the plethora of information about the pharmacology of amphibian natural products [17], [25], [32], rigorous demonstrations of the ecological functions of these metabolites are generally limited to anecdotal and observational accounts of prey distastefulness and predator resistance.

Like *Taricha* newts, the eastern newt (*Notophthalmus viridescens*) is assumed to deter predators by secreting TTX [27], [33], [34], with all life-history stages reportedly unpalatable to a variety of vertebrate and invertebrate predators [35], [36]. Despite the number of studies involving eastern newt defenses, the majority of the work has consisted of observational and anecdotal accounts that lacked proper statistical controls and sufficient replication [35], [36]. Earlier studies also often used consumer of questionable ecological relevance (e.g., farm chickens [37]). .Several studies of chemical defenses in *N. viridescens* used intraperitoneal injections of newt skin, ova, or purified toxin [38], often to the exclusion of predation bioassays with live prey (but see [29], [39], [40] for bioassays with a different newt species). Unfortunately, injections remove the effects of predator choice and physiological (e.g., salivary proline-rich proteins or cytochrome p450 enzymes) and/or behavioral detoxification methods (e.g., selective feeding on undefended tissues) that might occur during prey handling or digestion [41].

The lack of rigorous research on the chemically-mediated predator-prey interactions involving *Notophthalmus viridescens* is surprising given that eastern newts are thought of as keystone predators that regulate the diversity and abundance of larval anurans, aquatic invertebrates, and the ecosystem functions of some freshwater environments [42]–[44]. Therefore, we designed a series of experiments testing the hypothesis that *N. viridescens* are unpalatable to ecologically relevant generalist predators because of noxious skin secretions. Using laboratory-based feeding assays, we offered adult newts to predatory fishes (*Micropterus salmoides, Lepomis macrochirus*), crayfish (*Procambarus clarkii*), and bullfrogs (*Lithobates catesbeianus*). These were followed by assays evaluating the palatability of different newt tissues and different life-history stages (i.e., eggs, larvae, adults). Bioassay-guided fractionation was used to test the hypothesis that eastern newt defenses are chemically based. After determining that fishes largely avoided newts, we tethered adult newts in ponds with and without predatory fish to test the hypothesis that the risk of predation for amphibians—even chemically-defended species—is higher in permanent ponds containing fishes [10].

## Materials and Methods

### Ethics Statement

All procedures were approved by the Institutional Animal Care and Use Committee at the Georgia Institute of Technology under permit #A08047. Collections were under the auspices of National Park Service Permit #48516 and Georgia Department of Natural Resources permit #29-WBH-08-185. Permissions to work on private lands were obtained verbally from each land owner.

The eastern newt (*Notophthalmus viridescens* Rafinesque, Salamandridae) is one of the most widely distributed salamanders in North America [45] and occupies lentic environments across the spectrum from temporary to permanent water bodies. Shallow wetlands (≤1 m) with aquatic vegetation (e.g., *Chara, Utricularia spp.*) are ideal habitats, but large population densities (ca. 5 adults/m) can also occur in lakes, beaver impoundments, vernal pools, ponds and roadside ditches [45], [46].

*Notophthalmus viridescens* secrete tetrodotoxin (TTX) [27], [34], which could serve as a chemical defense against predators. Pharmacologically, the mechanism behind TTX toxicity is well described [47]; it is a neurotoxin that blocks nerve and muscle conductance through selective inhibition of sodium channels [47], [48]. Concentrations of TTX are greatest in the red eft stage, followed by adults, eggs, and finally larvae [27], [49], [50]. There are natural history and observational reports of newts being distasteful or toxic to predators (e.g., [35], [36], [51]), but statistically rigorous evaluation of this for *N. viridescens* are not available, as is the case for a surprising number of amphibians [52].

Adult *Notophthalmus viridescens* were collected by seine or dipnet from Georgia ponds in Walker, Rabun, and Athens-Clarke counties (Georgia Department of Natural Resources permit #29-WBH-08-185, with additional verbal permissions from each land owner). Newts were housed in aquaria with dechlorinated tap water at the Georgia Institute of Technology and fed fish pellets (African Cichlid Attack, HBH Pet Products, Springville, UT, USA) and live mealworms (*Tenebrio molitor*). Oviposition was encouraged by providing pieces of the aquatic macrophyte *Myriophyllum aquaticum* as a substrate. Eggs were maintained individually in 12×12×10 cm plastic containers holding dechlorinated tap water (6 cm depth) that was changed biweekly. After hatching, neonate larvae were fed freeze-dried copepods (Cyclop-eeze, Argent Chemical Laboratories, Redmond, WA, USA) three times a week.

To assess newt palatability to co-occurring consumers, we used adult largemouth bass (*Micropterus salmoides*; 15–20 cm standard length [SL]), juvenile bluegill sunfish (*Lepomis macrochirus*; 2–4 cm SL), the crayfish *Procambarus clarkii* (9–12 cm total length), and adult bullfrogs (*Lithobates catesbeianus*; 12.5–18 cm snout-vent length). Largemouth bass consume amphibians across all life-history stages [12], [53] and were collected from the Chattahoochee National Recreation Area (Atlanta, GA, USA; National Park Service Permit #48516). Bluegill are generalist consumers that can heavily impact amphibian communities [14], [54]. Our young-of-year bluegill were acquired from the Walton Fish Hatchery (GA Dept. of Natural Resources (DNR), Walton County, GA). We used juveniles because they require less food to obtain an observable response, thus minimizing the amount of newt assay materials required. Crayfish are generalist consumers that can dramatically alter the structure of freshwater amphibian communities through both predation and aggression [55]–[57]. They are not gape-limited and can prey on large adult salamanders as well as eggs and larvae. Adult crayfish were collected from the Clayton County Water Authority's experimental wetlands (GA). Bullfrogs consume insects, snakes, small mammals, fish, and other amphibians [58], [59]; ours were purchased from Niles Biological Supply, Inc. (Sacramento, California, USA). Bass and bullfrogs were individually housed in 76 L aquaria and maintained on a diet of earthworms (*Lumbricus terrestris*) or crickets (*Acheta domesticus*), respectively. Bluegill were housed in 38 L aquaria and maintained on a diet of fish pellets (African Cichlid Attack, HBH Pet Products, Springville, UT, USA), while crayfish were housed individually in 12×12×10 cm plastic containers within a recirculating freshwater table and fed herbivorous fish food.

For assays assessing palatability of adult animals, we used mole salamanders (*Ambystoma talpoideum*) as palatable alternative prey because they often co-occur with eastern newts and are equivalent in swimming ability and size [45]. All *A. talpoideum* were collected from the University of Georgia's Whitehall Forest. For assays assessing palatability of larval newts, we used larval spotted salamanders (*Ambystoma maculatum*) as alternative prey because they frequently share fishless ponds with both larval and adult eastern newts [45]. To obtain *A. maculatum* larvae, we collected two egg masses from a spring-fed pool in Palmetto, GA, maintained eggs in the laboratory until they hatched, and raised the larvae on freeze-dried copepods until they were used in experiments.

### Laboratory feeding assays

We offered consumers newts or similar control foods to determine the relative palatability of newts to consumers. We simultaneously offered an adult newt and a paedomorphic *Ambystoma talpoideum* to 11 largemouth bass, each housed in a separate aquarium. Prior to the assay, bass were fed one large earthworm to ensure feeding choices did not reflect starvation. The bioassay was initially conducted for 14 h (i.e., overnight, because largemouth bass are crepuscular feeders [53], [60]). After 14 h, each replicate tank was thoroughly inspected and the presence or absence of each salamander was noted. We then extended the bioassay for a total of 72 h to observe whether hunger would overwhelm any initial distastefulness; however, results at 14 h and 72 h did not differ. We used a one-tailed exact McNemar test for paired samples (exact2×2 package [61], *v.* 1.1) to assess differences in survivorship between the two salamander species with the *a priori* hypothesis that *N. viridescens* would be less palatable than the *Ambystoma* alternative. All statistical analyses were performed in *R* [62], *v.* 2.121.

For assays with crayfish, consumers were acclimated for 48 h in glass aquaria (51×27 cm) with 6 cm of water depth. Immediately prior to the bioassay, 10 newts and 10 *A. talpoideum* were euthanized by immersion in a 250 mg/L solution of MS-222 (tricane methylsulfonate) for 10 min followed by several rinses with deionized water. This minimized prey suffering and removed any differential escape behaviors, allowing a test of palatability alone. It is possible that MS-222 alters prey palatability, but unlikely given the compound's high solubility in water. Our subsequent and extensive rinses following death should have removed the compound, and a pilot study revealed no detectable affects on crayfish feeding. We simultaneously offered crayfish one newt and one *A. talpoideum*; after 18 h, both prey choices were removed and frequency of consumption assessed with a one-tailed exact McNemar test.

If toxin production in newts serves primarily as a defense against predation (instead of against microbes or other pests), then one might expect defenses to be preferentially allocated to body parts that are most exposed to consumers (i.e., dorsal skin). Young-of-year bluegill were used for assessing the palatability of differing newt body tissues. Six adult newts were euthanized, carefully skinned to keep the dorsal and ventral skin separate, and the internal organs then removed. The newt tissues (dorsal skin, ventral skin, and viscera) were cut into small pieces approximately 5 mm and each tissue type pooled for the bioassay. Individual bluegill were first offered a control food pellet (African Cichlid Attack, HBH Pet Products, Springville, UT, USA) to assure they were feeding. If the pellet was eaten, that fish was then offered one of the three treatment tissues. If the treatment food was rejected, the fish was offered a second control pellet to ensure the rejection response was not due to satiation. Separate fish were used for each body part assay to assure independence among consumers (dorsal skin: *n*  =  15, ventral skin: *n*  =  17, viscera: *n*  =  16). The frequency of acceptance for each treatment relative to the first control pellet was analyzed separately for each tissue type with a McNemar test. The relative palatability across newt tissue treatments was then analyzed using a likelihood-ratio test from a generalized linear model assuming binomial errors using the car package [63], *v.* 2.0–9. We conducted post-hoc pairwise comparisons with multiple Fisher's exact tests followed by Holm's sequential Bonferroni correction.

In a final assay with bullfrogs, an adult newt and a *Procambarus clarkii* crayfish (as a palatable alternative) were simultaneously offered to seven individually housed frogs. We used crayfish as alternative prey because we were unable to obtain appropriately sized *Ambystoma talpoideum* when this experiment was conducted. Before the assay, each frog received three crickets to ensure their responses were not starvation-induced. After prey were exposed to frogs for 24 h, each tank was thoroughly inspected for uneaten individuals and a one-tailed exact McNemar test was used to assess differences in survival between the two prey.

The above assays determined that newts were distasteful to fishes and crayfish, so we decided to also test the palatability of other life stages of *Notophthalmus viridescens* because it has been hypothesized that juvenile stages that must under-go dramatic developmental shifts cannot be chemically defended like adults [64], but this is not the case for marine species [65]. Because we were limited by the number of newt ontogeny available, we only used bluegill as assay predators, in part because juvenile fish were often found in vegetation occupied by newt embryos and larvae; crayfishes never were (Z.M., personal observation). Bluegill (*n*  =  10) were offered eggs of *N. viridescens* along with a control food pellet (African Cichlid Attack, HBH Pet Products, Springville, UT, USA) in a paired-choice feeding assay identical to the bluegill bioassay with adult newt tissues. Bluegill were first offered a food pellet followed by a newt egg. If the egg was rejected, a second control pellet was offered to assess whether that fish was satiated. We considered an egg rejected if the fish struck the egg, spit it out, and then ignored it.

To determine whether newt hatchlings were distasteful to fish, we offered bluegill a neonate newt larva and an *Ambystoma maculatum* hatchling in a paired-choice assay (*n*  =  13). We randomized the order of treatment that each fish received. Spotted salamander neonates are slightly larger on average than *N. viridescens* hatchlings (12–17 mm and 7–9 mm respectively; [45]), but juvenile bluegill showed no size-based attack preference and pursued both larval species equally. Because the salamanders did not hatch simultaneously, the bioassay was staggered over two days, ensuring that no larva was over 1 d old. No bluegill individual was used more than once. Tests with both eggs and hatchlings were evaluated via one-tailed exact McNemar tests.

Because adult *Notophthalmus viridescens* were consistently rejected by bass and crayfish, we evaluated whether rejections were (1) due to chemical deterrents, and (2) might be explained by the presence of TTX with bioassay-guided fractionation. To obtain newt crude chemical extracts we euthanized and eviscerated 10 adult newts, macerated non-viscera tissues in a blender with methanol and water (7∶3 v∶v), let this extract overnight, then successively extracted for two hours in methanol and dichloromethane (1∶0 v∶v, 1∶1 v∶v, and 0∶1 v∶v). Solvents were subsequently removed by rotary evaporation and the extract was then resuspended in methanol for bioassays. We gutted the newts because chemical defenses occur primarily in the skin [26] and because our previous predation assays indicated a rapid rejection that suggested metabolites are presented on newt exteriors.

To prepare test foods the extract was incorporated into a gel-based artificial food constructed with freeze-dried and finely ground frog legs, sodium alginate (27% by dry mass; Sigma-Aldrich, St. Louis, MO, USA) and 25 µg of red food coloring for visibility. The method for control foods was identical, including the solvent, but lacked the newt extract. Extracts were tested at double their yield (by volumetric equivalent) to offset loss due to extraction inefficiencies and chemical decomposition (see [66] for an example of such losses). This gel was loaded into a small syringe, squeezed into lines on a glass petri dish, and misted with a hardening solution of 0.25 M calcium chloride. After ca. 1 min, the gel was cut into bite-sized pellets. This method resulted in frog “noodles” with the consistency of cooked pasta (for an overview, see [67]). These treatment foods vs. identical control foods (but without the newt extract) were then offered to juvenile bluegill. Following confirmation of deterrent activity, the remaining crude extract was partitioned between ethyl acetate (lipids) and distilled water (the polar fraction), and fractions were tested separately for deterrent activity using the methods described above except that the polar fraction was resuspended in distilled water.

For the feeding assays, juvenile bluegill were held individually in 38 L aquaria divided across the middle with mesh so that each aquarium held two bluegill (one on each side). Prior to the bioassay, each fish was fed two food pellets to ensure feeding responses were not starvation-induced. In the afternoon each individual fish—assigned to treatments at random and interspersed spatially—received either a pellet containing newt extract or a control pellet and were monitored for acceptance or rejection of the treatment. No fish was used more than once. We determined statistical differences between experimental extract treatments and control treatments with a one-tailed Fisher's exact test.

### Predator avoidance conditioning

We wanted to evaluate how quickly naïve fish would learn to avoid food containing a range of TTX concentrations commonly found in eastern newts. Whole animal concentrations of TTX reported for *Notophthalmus viridescens* range from <0.15 to 146 µg/g of wet mass [34], [49]. Therefore, over 6 d, we exposed naïve bluegill to artificial foods reconstituted with one of five TTX concentrations: 0, 10, 20, 40, and 80 µg/g of TTX (*n*  =  20/treatment). The highest concentration we used (80 µg/g TTX) was only 55% of the highest concentration reported for *N. viridescens* in the wild. For each TTX treatment, tetrodotoxin citrate (Ascent Scientific, Princeton, NJ, USA) was reconstituted into a gel-based artificial diet after resuspension in distilled water using the methods described above. The control pellets were constructed identically but lacked TTX.

Prior to the experiment, juvenile bluegill were acclimated in divided aquaria (one fish per side). Each morning they received two food pellets to ensure feeding responses were not starvation-induced. In the afternoon an individual received one of the five treatments mentioned above. Fish were assigned to treatments by random interspersion. Over 6 d, we monitored whether fish ate the offered pellet or rejected it. One fish in the 10 µg/g treatment and two fish in the 80 µg/g treatment refused to feed throughout the experiment and were excluded from the analysis. The data were analyzed with a generalized linear mixed model (GLMM) fit by Gaussian Hermite approximation (nAGQ  =  15) and binomially distributed errors using the lme4 package [68], *v.* 0.999375-37. We started with a saturated model and used Akaike's Information Criterion [69] to remove all parameters but treatment and day as fixed factors and fish ID as a random factor then evaluated significance with likelihood ratio tests.

### Field predation experiment

We quantified the relative risk of predation for eastern newts in field habitats with and without fish by tethering adult *Notophthalmus viridescens* in paired, permanent ponds (*n*  =  6 pairs of ponds; 9–12 newts per pond) for 18 h in September of 2009—for each pair of ponds, one pond contained predatory fish (e.g., largemouth bass, bluegill) while the other pond lacked fish. We used permanent ponds because (1) *N. viridescens* often reach their greatest abundances in permanent fishless ponds [45], (2) to minimize any differences associated with the abiotic impact of drying (e.g., salinity, conductance, dissolved oxygen), and (3) the southeastern US was recovering from the effects of a severe drought and there were few ephemeral ponds still holding water at this time. Ponds within a pair were located no more than 2 km apart, making the experiment tractable and minimizing variance due to location. All ponds were drag-seined and dipnetted to identify the fish species present. Additionally, many fish-containing ponds were stocked for public fishing by the Georgia DNR so species presence could be verified with DNR catalogs of fish presence.

Newts were tethered by fastening a nylon cable tie (100×2.39 mm) around the torso just anterior of the pelvic girdle Prior to securing the cable tie, we fastened approximately 3 m of clear 3.63 kg test monofilament fishing line through the tie's ratchet case. The cable tie was tightened until the newt could not slip out but was left loose enough for the animal to rotate freely and allow unconstrained movement. The remaining length of cable tie was then cut flush with the ratchet case. We secured the fishing line to the pond bank with a 23 cm metal stake driven into the pond bank. The newt was then placed at the water's edge and allowed to enter on its own.

Newts were spaced ≥6 m apart and their presence or absence was monitored every two hours. We recorded newt presence, survival, and injuries from failed predation attempts. Potential predators near each individual were also noted (e.g., ranid frogs, turtles, predacious invertebrates). Because individual newts within ponds may not be independent, we calculated the final percent survival for each pond at the end of the 18 h exposure and analyzed the data with a paired t-test, using each pond pair as the level of replication.

## Results

**Figure 1:**
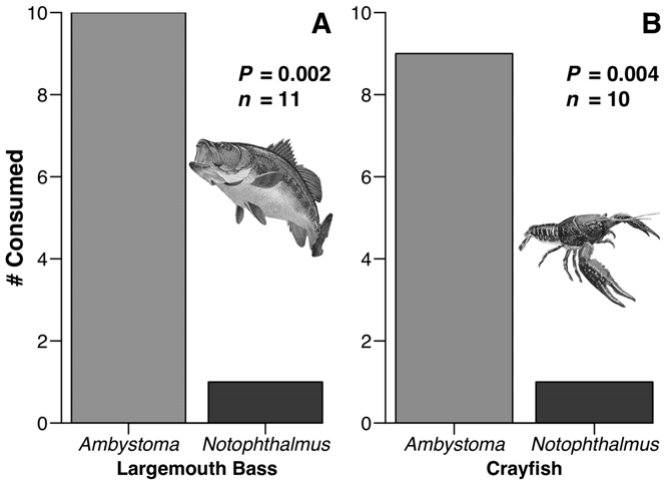
Consumption of the salamander *Ambystoma talpoideum* and the newt *Notophthalmus viridescens* in the laboratory. Both species were synchronously offered to either (A) largemouth bass or (B) the crayfish *Procambarus clarkii*. Statistical evaluation via a one-tailed exact McNemar test.

Newts were unpalatable to both largemouth bass (Figure 1A; *P*  =  0.002) and the generalist crayfish *Procambarus clarkii*, relative to *Ambystoma talpoideum* (Figure 1B; *P*  =  0.004). Both predators showed a 9–10X preference for *Ambystoma* over *N. viridescens*; i.e., nine and 10 *Ambystoma* were consumed for every newt by crayfish and bass, respectively. Bass took newts into their buccal cavity and immediately spit them out, followed by coughing and rapid operculation. A few bass repeatedly attacked the newts, but newts were rejected each time and no newts were killed, even when repeatedly attacked. Two newts had abrasions that suggested they had been chewed in the pharyngeal jaws before rejection; the injuries were not fatal. In contrast, no bass ever rejected an *A. talpoideum* salamander. The single newt consumed by a crayfish was eviscerated: its internal organs and much of the inner meat was consumed, but the dorsal skin was intact and unconsumed. Several other newts were missing toes and tail tips, suggesting they had been captured, sampled, and rejected. All but one of the *A. talpoideum* were almost completely consumed by the crayfish. Only the skull and spinal cord remained for nine out of 10 individuals.

**Figure 2:**
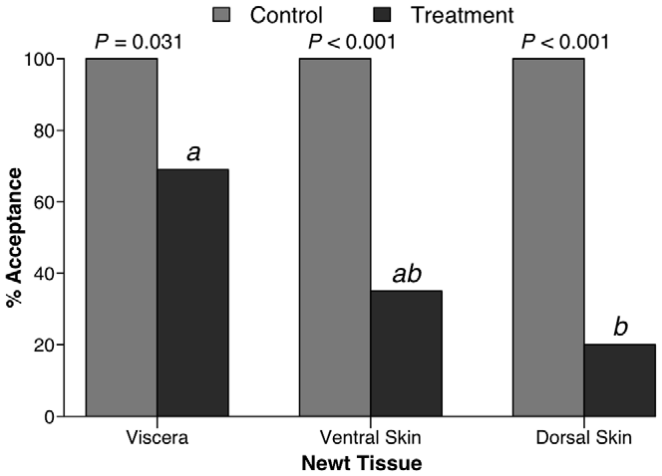
Consumption of different *N. viridescens* body tissues when offered to bluegill in laboratory feeding assays. Sample size (*n*) is below the bars for each paired assay. Letter designations above treatment bars indicate significant groupings via GLM (  =  8.241, *P*  =  0.016, *df* =  2,46) followed by pairwise Fisher's exact tests with Holm's sequential Bonferroni correction.

We used small bluegill to assess the palatability of different skin or body portions of newts. When compared to a palatable control food (100% consumed), newt viscera (75% consumed) were mildly deterrent (*P*  =  0.031), dorsal skin portions (20% consumed) were strongly rejected, while ventral skin (35% consumed) was rejected at an intermediate frequency (Figure 2; *P* ≤ 0.003). Newt tissues differed significantly from each other in palatability (GLM: *P*  =  0.016, *df*  =  2, 46), with dorsal skin being less palatable than viscera (Holm's-corrected Fisher's exact test: *P*  =  0.011) and ventral skin being intermediate between these tissues (Figure 2).

**Figure 3:**
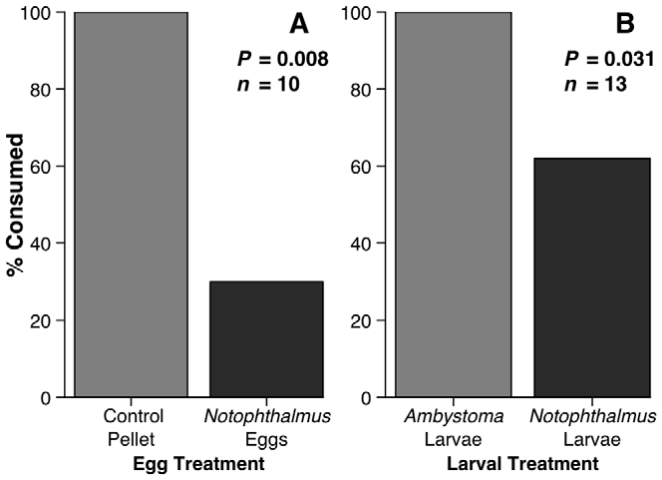
Consumption by juvenile bluegill of *N. viridescens* ontogeny in the laboratory. (A) Newt eggs versus equal sized control pellets and (B) neonate salamander larvae of *Ambystoma maculatum* versus newt larvae. P-values by one-tailed exact McNemar tests.

Eggs of *Notophthalmus viridescens* were also unpalatable to bluegill; they accepted only three of 10 eggs offered, but consumed all 10 of the paired control foods (Figure 3a; *P*  =  0.008). All fish that rejected eggs coughed and operculated rapidly. Of the seven rejected eggs, four remained viable and hatched live young.

When offered newt and *Ambystoma maculatum* hatchlings, bluegill consumed 13 of 13 *A. maculatum* but only 8 of 13 newts (Figure 3b; *P*  =  0.073). The five fish that rejected newt hatchlings took up to 2 min before spitting the larvae out, but the sampling by fish was fatal to the larvae.

**Figure 4:**
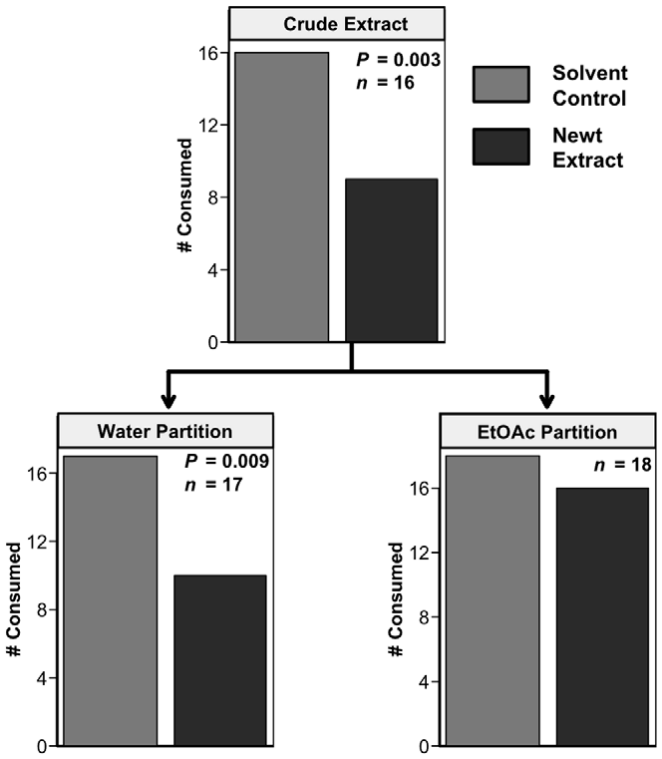
Bioassay-guided fractionation of the *N. viridescens* chemical extract when fed to bluegill in the laboratory. *P*-values are from one-tailed Fisher's exact tests.

To evaluate the role of chemical defense in *Notophthalmus viridescens* palatability, we extracted newts and assayed these extracts in feeding assays with bluegill. The crude extract deterred feeding by bluegill (Figure 4; *P*  =  0.003, *n*  =  16), as did the water-soluble (*P*  =  0.009, *n*  =  17) but not the lipid-soluble (ethyl acetate) partition of this extract (*P*  =  0.247, *n*  =  18). TTX and its derivatives, the bioactive compounds known to occur in newt skin, would have been in the water-soluble fraction.

**Figure 5:**
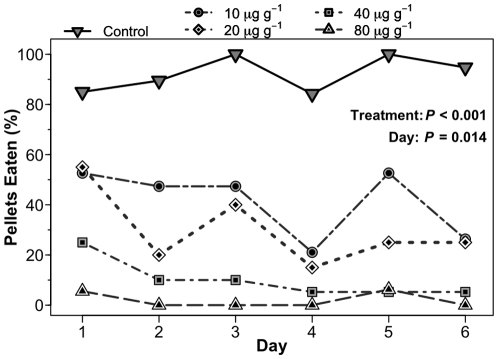
Percent of TTX-containing food pellets eaten by bluegill over 6 d of repeated feedings. Pellets were infused with different concentrations of TTX within the range found in newts from the field. *P* values are from likelihood ratio tests from a GLMM.

To assess how these TTX compounds would affect fish feeding as a function of dosage and fish experience with TTX, we fed naïve fish for six days on a palatable food treated with differing TTX concentrations that were within the range of natural concentrations documented for newts in the field. Both TTX treatment (*P*<0.001) and day (*P*  =  0.014) had significant effects on bluegill consumption (Figure 5). After six days of feeding exposure, acceptance of control pellets was 95%, acceptance of pellets with 10 or 20 µg/g TTX was ca. 25%, and acceptance of pellets containing 40 or 80 µg/g TTX was 0–5%. To put these concentrations into context, Yotsu-Yamashita & Mebs [50] reported a mean concentration of 16 µg/g (SD 6.3) TTX for adult newts in North Carolina. The highest TTX concentration known for eastern newts is 146 µg/g [34].

When adult newts were tethered in spatially paired fish-containing and fishless ponds in the field, newts experienced 62% (SD 19.7) mortality in fish-containing ponds, but only 40% (SD 24.1) mortality in fishless ponds (paired t-test: *P*  =  0.029). Many victims of predation had body parts cleanly bitten off which is consistent with turtle predation, not fishes like bass, and when checking tethers it was not uncommon to observe small turtles (Kinosternidae; approx. 5–8 cm TL) moving away from half-consumed newts as we approached. On several occasions, bullfrogs (*Lithobates catesbeianus*) had swallowed newts, and we saw these being regurgitated from the frog as we pulled the monofilament line to retrieve the newt. Although we focused on fish-containing and fishless ponds, it need not have been fish generating the predation differences we detected.

To see if adult bullfrogs readily fed on newts, we simultaneously offered seven bullfrogs newts and similar sized crayfish (*Procambarus clarkii*); other controls, such as *Ambystoma* salamanders, were unavailable at this time). After 24 h, all crayfish and six of the seven newts had been consumed (McNemar test: *P*>0.99). No frog exhibited visible signs of stress due to newt consumption.

## Discussion

Consumers have strong impacts on prey communities [1], [7], and this appears especially true for the amphibians in freshwater systems [42], [70]; one might therefore predict strong selection for prey defenses. Despite their small, soft bodies and high nutritional value, some species of frogs, toads, and salamanders commonly co-occur with predators, and this often correlates with the production of bioactive chemicals [17], [70]. Despite the broad assumption that these amphibians are chemically defended, there are few rigorous tests of amphibian chemical defenses using co-occurring consumers [40]. We therefore know little about the ecological efficacy of these compounds in deterring generalist consumers or the variation in effectiveness against different consumer types. Here we show that eastern newts, their early ontogeny, and especially their dorsal skin areas are distasteful to common aquatic consumers such as fish and crayfish (Figures 1, 2, 3), that fish avoid newts due to chemical deterrents (Figure 4), and that the compound TTX can produce this response at small portions of its reported natural concentrations (Figure 5). Yet some consumers, such as bullfrogs and possibly other reptiles and amphibians [31], [40], appear undeterred by TTX.

Bluegill were most deterred by newt dorsal skin (about 80% rejection), less by ventral skin (about 65% rejection), and even less by viscera (about 30% rejection; Figure 2), suggesting that chemical defenses are concentrated in those tissues first contacted by consumers. Hanifin et al. [71] found a similar gradient in the distribution of both TTX toxicity and the granular glands that secrete TTX in the skin of the newt *Taricha granulosa*. Distaste also varied with newt ontogeny. The fertilized eggs of *Notophthalmus viridescens* were unpalatable to bluegill (Figure 3a), and the few fish (*n*  =  3) that consumed eggs coughed repeatedly and rapidly operculated, suggesting that they were in distress or were attempting to “wash” the eggs of toxins prior to consumption [72]. Other amphibian eggs also appear chemically defended. Eggs of bufonid toads contain cardiotoxic bufodienolides [73], and *Taricha* newt eggs contain TTX [74]. Thus, despite the early hypothesis that toxins and early life stages were incompatible due to the difficulty of managing toxins during rapid developmental changes [64], chemical defense of eggs and larvae occurs among both marine [65] and terrestrial invertebrates [75]. Larval newts that hatched from distasteful eggs were consumed at somewhat higher rates than were eggs but at lower rates than the chemically benign larvae of spotted salamanders (Figure 3b). Given that amphibian embryos and neonates are especially vulnerable to predation [70], any defense at this stage is likely an adaptive advantage. We would have liked to include crayfish as well as bluegill in the ontogeny study; previous research demonstrated that invasive *P. clarkii* in California consume large amounts of *Taricha* embryos and larvae [55]. Unfortunately, we were limited by the number of eggs and neonate larvae we had available. However, if the results of Gamradt and Kats [55] are any indication, the eggs and larvae of *N. viridescens* are likely susceptible to crayfish predation, though the frequency of encounters between crayfish and eastern newt embryos and larvae remain an open question.

These results also show that chemically-mediated deterrence is quantitatively nuanced. Like the bluegill we used, potential consumers sampling chemically-defended prey make decisions regarding consumption based on compound concentration as opposed to presence alone (see also [40]). Despite the likelihood that TTX is the chemical responsible for most of the predator deterrence in eastern newts, other molecules could be involved. Most newt species produce tetrodotoxin derivatives, but we know much less about their efficacy and ecological impacts, or how the compounds interact with one another. Moreover, Yotsu-Yamashita and Mebs [50] found that TTX concentrations in some *Notophthalmus viridescens* efts were insufficient to explain the level of bioactivity to injected mice; the authors speculated on the existence of additional bioactive compounds, but they were not identified. We tried to further separate and purify the bioactive, water-soluble fraction shown in Figure 4 but lost deterrence following further purification steps, suggesting instability or synergistic effects that were lost as metabolites were separated. However, our results clearly show that generalist predators rapidly learn to avoid the consumption of food impregnated with relatively low concentrations of TTX (7–14% of the maximum reported concentration in *Notophthalmus* [34]), and most instantly rejected food containing 80 µg/g (55% of the maximum known natural concentration; Figure 5).

Despite the chemical distastefulness of newts to important aquatic predators like bass, bluegill, and crayfish (Figures 1, 2, 3), newts experienced mortality of about 60% in fish containing ponds and of about 40% mortality in fishless ponds when tethered in the field. Although we chose fish-containing and fishless ponds for these contrasts, it need not be fish that directly produced this difference in survivorship.

There are several potential explanations for the disconnect between fish rejection of newts in our laboratory bioassays and the considerable mortality of newts in fish-containing ponds. First, tethering newts constrains their movement, possibly interfering with predator evasion. It is possible that distastefulness causes initial hesitation by many consumers and facilitates newt escapes into refuges. Despite this potential artifact, our tethering experiment still demonstrated differences in mortality between fish-containing and fishless ponds. Second, in most cases we had to infer predation from presence/absence data. Yet predation clearly caused the loss of numerous newts; some *N. viridescens* had skin abrasions indicative of sampling within the pharyngeal jaws of largemouth bass followed by rejection–similar abrasions were observed on newts following the laboratory feeding assays with bass. Other newts were pulled from bullfrog stomachs as we retrieved the tether, and some were bitten in half, apparently by turtles (i.e., *Sternotherus* and Kinosternon spp.) that we observed swimming away from these newts as we approached. These observations suggest that our initial laboratory assays did not encompass some of the relevant predators of newt adults. Hurlbert [36] noted that snapping turtles (*Chelydra serpentina*) and painted turtles (*Chrysemys picta*) would consume *N. viridescens*, and several of the partial newt bodies we recovered looked as if they had been cut by the sharp beaks of turtles. Additionally, Brodie [35] observed that bullfrogs would eat eastern newts and their efts without distress, and Hurlbert [36] kept a large bullfrog for months on a diet of *N. viridescens*. Although these earlier studies lack replication, they are consistent with our findings that bullfrogs consume newts at the same frequencies as prey lacking noxious chemicals (*p*>0.99).

Because turtles and bullfrogs co-occur in ponds with predatory fishes [58], [59], we hypothesize that the interactions among bullfrogs, turtles, predatory fishes, and chemically-defended amphibians like eastern newts may represent an example of “apparent predation,” to borrow from the apparent competition paradigm of Holt [76], [77]. In apparent competition, one prey species is negatively affected by the presence of other prey species because of the indirect attraction of shared predators. The resulting patterns are identical to those produced by interspecific competition. Here we argue that fish—the limiting predators for many prey species in permanent lentic habitats [10]—reduce the densities of newts, not through direct consumption, but indirectly by excluding the predators of larval bullfrogs (e.g., dragonfly larvae). Once bullfrog larvae reach adulthood, the adults then directly consume adult newts. Thus, while fish presence is correlated with increased newt predation and lower newt densities in the field [12], [54], it may be that fish are controlling important predators of bullfrog larvae, allowing higher densities of adult bullfrogs, and it is the bullfrogs that are consuming adult newts (i.e., a case of predator-predator facilitation).

Consistent with this hypothesis, when Werner and McPeek [14] manipulated the presence or absence of bluegill, bullfrogs survived only in ponds that contained bluegill. The authors deduced that, in the absence of bluegill, invertebrate and salamander predators were consuming all bullfrog larvae. When bluegill were present, they excluded these predators and facilitated the survival of the bullfrog larvae that bluegill, bass, and other predatory fishes find noxious but apparently not toxic [78]–[80]. Smith et al. [54] found similar results when they manipulated bluegill densities. Additionally, Adams et al. [81] found that dragonfly nymphs reduced introduced bullfrog larvae by 100% in Oregon unless non-native sunfish were introduced to deplete dragonfly densities.

Our results show that the newt *Notophthalmus viridescens* is unpalatable to fishes and a crayfish, and that this unpalatability is chemical in nature, is concentrated in exterior dorsal skin, and is likely due to TTX or related secondary metabolites. However, this chemical defense is ineffective against bullfrogs (and possibly turtles), allowing considerable consumption of tethered newts in the field. Consistent with previous work, we found that newt mortality was less in fishless than in fish-containing ponds in the field. Yet our data suggest that the mechanism by which fishes determine the distribution and abundance of eastern newts and other chemically-defended prey species may be indirect rather than direct and appears more complicated than generally appreciated.
